# Physicochemical and Sensory Evaluation Data to Drive the Development of a Green Chili Pepper Hot Sauce from Unexploited Raw Materials

**DOI:** 10.3390/foods12193536

**Published:** 2023-09-22

**Authors:** Paula Torán-Pereg, Shuyana Deba-Rementeria, Olaia Estrada, Guillermo Pardo, Laura Vázquez-Araújo

**Affiliations:** 1 BCC Innovation, Technology Center in Gastronomy, Basque Culinary Center, 20009 Donostia-San Sebastián, Spain; 2 Basque Culinary Center, Faculty of Gastronomic Sciences, Mondragon Unibertsitatea, 20009 Donostia-San Sebastián, Spain; 3 Basque Centre for Climate Change (BC3), 48940 Leioa, Spain

**Keywords:** Espelette, new product development, food waste, fermentation, Napping^®^

## Abstract

The present study shows the set of analyses conducted during the development of a hot chili pepper sauce to valorize green peppers usually discarded in the Espelette region (France). A traditional production process was used as the inspiration for product development, and two different fermentation processes were assessed and characterized by measuring pH, sugar content, instrumental color, volatile composition, and conducting sensory (discriminant test) and microbiological analyses (total plate count). Significant differences were observed among pepper mash samples with respect to their physicochemical characteristics, but the products were considered similar from a sensory standpoint. Both sensory and physicochemical tests suggested that the ingredients added to make the sauces were determinant and had a higher impact on the organoleptic profile of the final product than the fermentation process. Finally, a Napping^®^ test was conducted to determine the attributes that could differentiate the product from the hot sauces found in the current market. The results of the present research allowed the optimization of the elaboration process of the new product, saving time and ingredient costs. The procedures shown in the study could be used as an example of a new product development process in which physicochemical and sensory data are collected and used for decision making.

## 1. Introduction

The geographical origin of peppers (*Capsicum* spp.) has been reported to be tropical America [1] but, thanks to its adaptability to different environments, this plant has spread across different parts of the world, mainly warm-temperature regions [2,3]. Over 20 to 30 species of pepper have been identified, including five domesticated species: *C. annuum*, *C. chinese*, *C. pubescens*, *C. frutescens*, and *C. baccatum* [4,5]. This huge biodiversity has given rise to a great variety of fruits with unique morphologic (e.g., shape and size) and organoleptic characteristics (e.g., color, flavor, pungency), and different gastronomic cultures have developed diverse products and applications from peppers (e.g., fresh, dried, processed as spices, pickled, pastes, sauces, or roasted).

Espelette peppers (*C. annuum* var. Gorria) are one of the most renowned peppers in the Basque Country. Their growth in specific natural conditions (e.g., landscape, weather, soil, plant varieties, etc.), together with localized human factors (e.g., habits, culture, specific know-how, etc.), allowed the registration of Espelette peppers as having Protected Designation of Origin (PDO *Piment d’Espelette—Ezpeletako Biperra*, in the French and Basque languages, respectively) status in the European Union in 2012 [6]. The specifications of this regulation indicate that at least 80% of the surface of the pepper must be red when harvested to belong to the “Espelette pepper” PDO [7], among other requirements. At the end of the harvesting season (before 1 December), when the sun is less intense and the plant is at the end of its vegetative cycle, some fruits are not completely ripened (still being green) and are characterized by their intense spiciness. Because of the lack of commercial outlets, tons of green fruits remain on the plant and end up being discarded.

Gastronomy has been proven useful for identifying and up-cycling discarded foods, transforming them into new ingredients with higher values and acceptance [8,9,10,11]. Different Asian, African, and American food cultures are characterized by chili peppers, demonstrating that spicy food is particularly popular in some gastronomies [1,12,13,14]. Although traditional Basque cuisine is not recognized as being spicy, hot sauces are spreading to food cultures in which this flavor characteristic is not typical. These sauces are market-oriented products of which consumption is globally increasing, probably due to factors such as increases in trade, migration, travelling, and the current trend of consuming ethnic foods in the western regions [13,15,16,17].

The production processes of some of these sauces (e.g., tabasco, sriracha) include a fermentation process of the peppers, which can last from two weeks to three years [18,19]. In this fermentation, peppers are mashed together with salt, generating a selective environment in which the growth of lactic-acid bacteria (LAB) is favored [20]. The selected microorganisms metabolize the different components of the food matrix and produce organic acids, volatile compounds, etc., which bring the distinctive organoleptic properties and contribute to the nutritional profile of the resulting product, increasing its value and driving consumers’ acceptance [21]. Fermentation processes can be classified in two main categories: spontaneous fermentations and driven fermentations. The first one, which has been widely used in homemade and culinary fields [22,23], results from the competition between the autochthonous microorganisms (yeasts, fungi, aerobic and anaerobic bacteria) present in the product, but these spontaneous fermentations may easily fail due to contamination [24]. Driven fermentations require the addition of a specific starter (e.g., a lactic acid bacteria strain), ensuring a more standardized product. Previous research has reported that the use of autochthonous isolated bacteria is a preferable process to drive a specific fermentation, because these bacteria may be better adapted to the raw material [25,26]. To finish the hot pepper sauce making process, after the fermentation, the pepper mash is generally mixed with other ingredients such as vinegar, garlic, sugar, water, vegetables, etc., to obtain a specific final product [18]. Considering all the ingredients, and the complexity of flavor of this product category, the role of fermentation could be linked to the preservation of the raw materials more than bringing specific sensory notes to the final product.

The aim of this study was to develop a green chili pepper hot sauce to valorize the unexploited green peppers of the Espelette region, using sensory and physicochemical data to propose an optimized process and successful product. Different methods, that allowed characterization of the process, prototypes, and products, were used in the present research, including sugars determination, instrumental color, volatile composition, discriminant sensory tests, and Napping^®^.

## 2. Materials and Methods

### 2.1. Reagents

Ultrapure water (Type I, 18.2 mΩ-cm) was from an Elga Purelab Flex 3 (ELGA LabWater, High Wycombe, UK). Sodium chloride (99.9%) was supplied by VWR (VWR Inc., Darmstadt, Germany). Analytical quality grade standards of glucose (1000 mg L^−1^), fructose, and sucrose were supplied by Sigma-Aldrich (Merck KGaA, Darmstadt, Germany). Sodium hydroxide solution (1 mol L^−1^) and sodium acetate anhydrous (>99%) (HPLC mobile phase) were supplied by Panreac AppliChem (Panreac AppliChem, Barcelona, Spain) and Sigma-Aldrich (Merck KgaA, Darmstadt, Germany), respectively. Peptone saline solution, Man, Rogosa and Sharpe (MRS) agar and nutrient agar were from VWR (VWR Inc., Darmstadt, Germany). The alkane standard mixture for gas chromatography–mass spectrometry compound identification was purchased from Sigma-Aldrich (Merck KgaA, Darmstadt, Germany).

### 2.2. Pepper Samples and Sauce Making Samples

**Figure 1:**
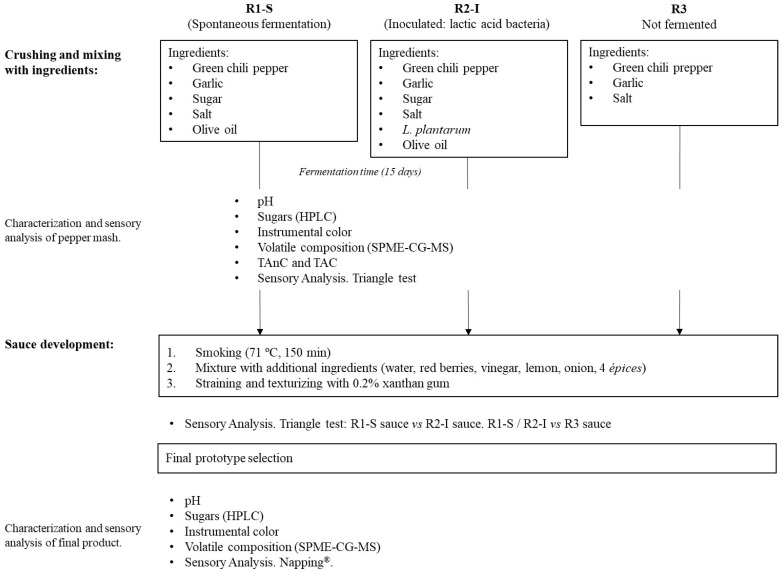
Scheme of the processes studied during the present research.

Green peppers (*C. annuum* var. Gorria) were obtained from a local producer from Espelette (France). The samples were harvested during the last week of November 2021, washed in tap water, and stored in a freezer (−20 °C) until processing. The list of ingredients and summary of the processes used to make and analyze the 3 prototypes is shown in Figure 1; the main differences among them were linked to the fermentation process, to assess the impact of this stage in the final product.

The peduncles of the fruits were removed, and the rest of the peppers were mashed in a blender with different ingredients (Thermomix TM6, Wuppertal, Germany). The samples to be fermented (R1 and R2) were transferred to sterilized glass jars (500 g/jar); one batch was left to ferment using the autochthonous microorganisms (spontaneous fermentation, R1-S), and the other one was inoculated with *Lactobacillus plantarum* Harvest LB-1 (0.08% as suggested by the producer of the starter; Chr Hansen, Hvidovre, Denmark) (R2-I). *L. plantarum* was chosen due to being one of the multiple lactic-acid bacteria found in peppers, which has been previously studied in pepper fermentation [25,27,28]. The samples were covered with 20 mL extra virgin olive oil (Urzante, Tudela, Spain) to avoid the surface coming into contact with the oxygen of the head space, sealed tightly, and stored at room temperature (21–24 °C) for 15 days. One of the samples (R3) was directly processed without fermentation into the final product; therefore, sugar was not added to this sample, and it was considered an example of the “original mash” at time 0 days. The three mash samples (R1-S, R2-I, and R3) were smoked (at 72 °C for 150 min) and mixed with other ingredients to finish the formula of the sauce (Figure 1). Then, samples were strained and thickened with xanthan gum (0.2%), obtaining 3 different prototypes of sauce.

### 2.3. Characterization of the Fermentation Process and Products: Green Chili Pepper Mashes and Sauces

The original mix, the fermented mash, and the final products were analyzed using different physicochemical analyses. Microbiological analyses were performed to verify that the fermentation procedures used in R1-S and R2-I were driven by different microorganisms and, therefore, that using the starter added in R2-I could result in a different product. All analyses were run in triplicate. In addition, sensory tests were performed to determine the differences among samples.

#### 2.3.1. Determination of pH

The pH was determined at 20 ± 0.5 °C using a digital pH meter (Crison Basic 20, Crison instruments, Barcelona, Spain) calibrated with standard buffer solutions.

#### 2.3.2. Microbiological Analysis

The mash samples were diluted in tenfold series in saline peptone solution (0.85% NaCl and 1% peptone) and plated onto different culture media to quantify the presence of different microorganisms using a traditional Plate Count Agar (PCA) method (ISO 4833-1:2013) [29]: nutrient agar for total viable colonies in aerobic conditions (30 °C for 48 h) and MRS agar for lactic acid bacteria (37 °C for 72 h, in anaerobic conditions).

#### 2.3.3. Determination of Sugars

Sugars determinations were conducted as reported by Razola-Díaz et al. [30] with some modifications. Freeze-dried mash samples were diluted 1:100 (*v*/*v*) with ultrapure water at 60 °C, then shaken for 30 min, and centrifugated at 2367× *g* for 10 min. The supernatant was filtered using a 25 mm, 0.45 μm nylon VWR^®^ Syrenge Filter (VWR Inc., Darmstadt, Germany), and 50 μL were injected in the same equipment as Deba-Rementeria et al. [8], using the same column and conditions indicated by these authors. Calibration curves for glucose, fructose, and sucrose, were used to quantify the presence of these compounds, identified by retention time.

#### 2.3.4. Instrumental Color

The color of the samples was measured with a Chroma Meter CR 400 (Konica Minolta, Inc., Osaka, Japan). CIE L*a*b* color space, an illuminant D65, and a 10° observer were used as references. Data were expressed with the L*, a*, and b* values and then, chroma [C* = (a*^2^ + b*^2^)^1/2^], hue angle [H = tan^−1^(b*/a*)], and total color change [ΔE* = [(L* − L_0_*)^2^ + (a* − a_0_*)^2^ + (b* − b_0_*)^2^]^1/2^] were calculated. Ten measurements were made for each of the samples’ replicates.

#### 2.3.5. Analysis of Volatile Composition

The volatile compositions of the samples were determined by headspace solid phase micro-extraction (HS-SPME) using the same conditions, fiber, column, and equipment reported by Deba-Rementeria et al. [31]. A total of 0.5 g of freeze-dried sample was weighted and ultrapure water (10 mL) and NaCl (1.0 g) were added into the 40 mL vial with polypropylene caps and PTFE/silicone septa for the extraction and processing as reported by these authors. Retention indexes of a commercial alkane standard mixture (Sigma-Aldrich, Steinheim, Germany) were used to identify the compounds, as well as the NIST 17 Mass Spectral and Retention Index Libraries [32]. The identification was considered tentative when only based on mass spectral data; a linear retention similarity filter was set at ±10 units. The relative abundance of each compound was expressed as the percentage (%) of the total arbitrary area units.

#### 2.3.6. Sensory Analysis of the Samples

The protocol for the consumer study was approved by the ethics committee of Mondragon Unibertsitatea (IEB-20221115). All articles from the Declaration of Helsinki and the 2016/679 EU Regulation on the protection of natural persons regarding the processing of personal data and on the free movement of such data were met. The experimental procedure was explained to and a written consent indicating voluntary participation was obtained from each participant prior to beginning the study. The tasting sessions were conducted in a sensory lab with individual booths and controlled temperature and relative humidity (21 ± 2 °C; 55 ± 5% RH); the illumination was a combination of natural and nonnatural light (fluorescent). To determine if sensory differences could be perceptive between products, a triangle test was performed following the UNE-EN ISO 4120:2022 procedure, considering a = 0.05 and the recommended randomization design (ABB, AAB, ABA, BAA, BBA, BAB) [33]. A panel of 25 assessors trained for discriminant tests participated in the study and conducted a series of triangle tests in different sessions: (1) R1-S mash sample vs. R2-I mash sample; (2) R1-S sauce vs. R2-I sauce; and (3) R1-S and R2-I sauces vs. R3 sauce. Cream and breadsticks were provided for palate cleansing between samples.

After observing that the prototypes were considered similar from a sensory standpoint, a Napping^®^ test [34] was conducted with the R3 sample to identify similitudes and differences between the developed sauce and similar products found in the local supermarkets (13 samples; shown in Table 1). A total of 21 professional chefs/gastronomes participated in the session to ensure familiarity with the product category. Approximately 10 mL of each sample were served in 20 mL disposable cups coded with 3-digit random numbers. Participants were asked to smell and taste the sauces and place them in an A3 paper (297 × 420 mm), close together or further apart, depending on their similarities/differences. In addition, assessors were instructed to include descriptors for each sauce/group of sauces. Cream and breadsticks were provided for palate cleansing between samples.

### 2.4. Data Analysis

Physicochemical and microbiological determinations were analyzed using one-way Analysis of Variance (ANOVA) using “recipe” as factor, followed by post-hoc Tukey’s HSD test (α = 0.05). The results of the triangular test were analyzed as suggested by the UNE-EN ISO 4120:2008 procedure [33]; the significance was determined by considering the minimum number of correct responses. A multiple-factor analysis was carried out with the Napping^®^ test [35]. The results were analyzed using XLSTAT (Version, 2021.5, Addinsoft, Denver, CO, USA) [36].

## 3. Results and Discussion

### 3.1. Mash Characterizations: Role of the Fermentation Process

Table 2 shows the initial pH of the original green chili pepper mash without fermentation, corresponding to sample R3 (5.2), and both R1-S and R2-I mash samples after 15 days of fermentation, which had a significantly lower pH value (*p* ≤ 0.05). The inoculated sample (R2-I) reached a lower pH (3.7) than the spontaneously fermented sample (4.8). Similar results have been reported by other authors; Di Cagno et al. [25] showed that spontaneously fermented red and yellow peppers (*C. annuum* L.) had higher pH values (pH above 4.8) than those inoculated with autochthonous bacteria (*L. plantarum* PE21, *L. curvatus* PE4, and *W. confuse* PE36) (approximately 3.6). Aryee et al. [17] reported a decrease of pH from 5.24 to 4.87 after 14 days of fermentation in habanero peppers (*C. chinese*) fermented spontaneously in a brine with a 5% salt concentration. The pH of Tabasco pepper mash (*C. fructescens* grinded with 8% salt), changed from 4.7 to 3.9–3.7, depending on the material of the container (plastic and wood, respectively), after the first month of a spontaneous fermentation [37]. The results of the present study suggested that using a low salt concentration, together with the inoculation of a commercial *L. plantarum* strain, favored the pH decrease. These results support the use of microorganisms well-adapted to the medium to promote the fermentation process. Reaching low pH values is recommended to ensure food safety while avoiding refrigeration of the product, because shelf stable hot sauces must have a pH below 4.6 [38].

Significant differences were found in total aerobic counts (TAC) and total anaerobic counts (TanC) among the samples (Table 2). TAC significantly decreased during fermentation in both the R1-S and R2-I samples, probably due to the lack of a proper environment for aerobic microorganism development. TanC increased in both samples during fermentation, starting from 4.6 log CFU ml^−1^ and reaching a significantly different amount in R2-I T15 (8.8 log CFU mL^−1^), the sample inoculated with *L. plantarum*. No significant differences were found between the pepper mashes T0 and R1-S at T15, although a slight increase was observed after 15 days of fermentation. Di Cagno et al. [25] showed an increment in lactic acid bacteria counts (*L. plantarum*, *L. curvatus*, and *W. confusa*) in peppers fermented at 35 °C during 15 days in a brine (1% NaCl), passing from approximately 4 log CFU/g to 9 log CFU/g; these authors reported higher increments in the samples in which starters were used. An increase in LAB count was also reported by Janiszewska-Turak et al. [27], who studied the differences between a spontaneous fermentation of green peppers (*C. anuumm* L.) vs. a fermentation driven by an inoculated starter of *Levilactobacillus brevis*, *Limoslactobacillus fermentum*, and *L. plantarum*. Fermentation time was different between the present study and the one reported by Janiszewska-Turak et al. [27] (15 days and 7 days, respectively), which may have led to a different result than the one obtained in the present study.

All color parameters varied significantly (*p* ≤ 0.05) during the fermentation process (Table 3). Lightness (L*), the blue-yellow coordinate (b*), chroma, and hue increments were significantly higher in the R2-I mash sample. On the contrary, the red-green coordinate (a*) and the total color transformation, represented by ΔE*, were higher in the mash sample of the spontaneous fermentation (R1-S). The fermented samples’ colors were significantly different from the original mash, ΔE* being over 5 in both the R1-S (T15) and R2-I (T15) samples; ΔE* has been reported to show differences between treated and untreated samples when being over 0.5 in citrus products [31,39]. Janiszewska-Turak et al. [27] reported that, in general, the L*, a*, and b* coordinates increased during the fermentation of green peppers driven by different microorganisms, with a higher increase of L* in the samples fermented by *L. plantarum*, and a higher increase of a* in the spontaneously fermented samples.

The volatile compositions of the mash samples are shown in Table 4. In general, the volatile compositions of the mash samples were mainly represented by compounds found in garlic such as diallyl disulfide or allyl methyl trisulfide [40,41], and other compounds previously found in chili peppers (e.g., 2-Isobutyl-3-methoxypyrazine, several aldehydes and alcohols [42]). Significant differences were found among samples, suggesting that the fermentation process influenced the aromatic profile of the samples.

To assess if the differences found in the physicochemical compositions of the mashes were perceived by the human senses, triangle tests were conducted with a trained panel of 25 assessors. Results showed that no significant differences were perceived between fermented mashes (correct answers = 10; at least 13 correct answers were needed to consider samples to be significantly different considering a = 0.05), suggesting that the different processes led to products that could be considered different from a sensory standpoint. The pungency of the mash samples could have influenced the perception of the differences identified in their volatile compositions or sugars profiles. Therefore, because other ingredients would be later incorporated to finish the product, and these could have a significant effect on their perception, additional triangle tests were conducted to determine if the three samples of sauce prototypes were perceived as different. No significant differences were detected among sauce samples, suggesting that, in general, the ingredients had a higher effect on the flavor of the sauces than the production process. The results of the sensory tests leads to the conclusion that the fermentation stage, although typically conducted in other products of the same category [18,19], was unnecessary to develop the proposed new product of the present research, probably because the contribution of the fermentation to the organoleptic properties of the sauce was limited in this product.

The use of different sensory tests, such as discriminant tests, combined with physicochemical data has proven to be useful in the decision-making process of different product developments such as ice cream recipe reformulations [44], testing the application of de-greening treatments in citrus [45], or estimating the shelf lives of fish broth products [46]. The present study supports the need to use at least simple sensory tests, as well as physicochemical data, in the design process of a product.

### 3.2. Final Product Characterization

Table 5 and Table 6 show the physicochemical characteristics and volatile composition of the final sauce sample. The mixture of the mashes with the additional ingredients significantly modified their characteristics, resulting in what could be considered a kind of product with homogeneous sensory characteristics. The pH of the final product was significantly lower (*p* < 0.05) than the pH of the mashes, probably due to the addition of lemon juice, comprising 8%. The pH reached after adding this ingredient (pH = 3.3) ensured the absence of pathogen bacteria growth because it is under 4 [47], and supported the results of the sensory tests, confirming that the fermentation stage was not useful to differentiate the flavor of the product, nor was it necessary from a technical standpoint.

Sugars and color parameters were also heavily influenced by the additional ingredients. Although green chili peppers were the main ingredient of the sauce, the presence of other ingredients such as lemon juice and red fruits completely modified the sugar content and color of the final product, having higher a* coordinate values (redder than the mash samples) and lower b* coordinate values (bluer than the mash samples).

Finally, the volatile composition of the sauce was significantly affected by the presence of the additional ingredients (Table 6). Approximately 18 volatile compounds of the mash samples were also present in the sauce samples, but limonene and eugenol represented over 50% of the total area of the volatile profile of the sauce samples, suggesting the importance of the additional ingredients (lemon juice and 4 *épices*) in the aromatic profile of the product. The final sauce had over 45 different volatile compounds from different chemical groups (terpenes, benzene derivatives, sulfide derivatives, etc.) showing the complexity of the product and confirming the difficulty of perceiving the flavors coming from the potential fermentation stage.

**Figure 2:**
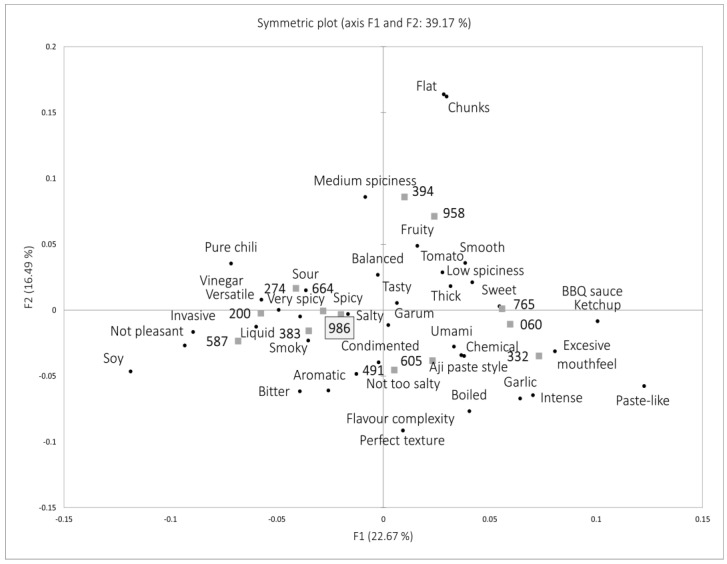
Results of the Napping^®^ test conducted with the samples listed in Table 1. Note: cubes refer to sauce samples and dots to the descriptors for each sauce/group of sauces.

With the aim of mapping the new product in the food market, and understanding the similarities and differences among competitors, a Napping^®^ test was also developed by food experts (chefs and gastronomes) who could aid in positioning and describing the new product. Figure 2 shows the symmetric plot of all the sauces listed in Table 1, explaining the 39% variability of the samples.

The sample developed during the present research was described as “sour”, “spicy”, “smoky”, or “balanced”, features shared with commercial sauces such as Valentina (664) and Tabasco chipotle (383), and that could be used to communicate its profile and encourage the selection of the developed product by consumers who prefer these attributes over other ones such as “sweet”, “dense”, or “bitter”, which were used to describe other products such as Spanish sriracha (765), Gochujang (332) and Soya-based chili sauce (587). Different studies have shown that the intensive consumption of spicy foods could favor the ability to discriminate flavors in this type of product [12,48]; therefore, it is possible that heavy consumers or sensory experts in the corresponding food category (hot sauces) could detect subtle differences among the products. Further studies should be conducted to determine if consumers from different food cultures (e.g., gastronomies with high vs. low use of spicy ingredients) have different discriminatory abilities for these hot sauces.

## 4. Conclusions

Results of the present research suggest that the fermentation stage of the production process of some hot chili pepper sauces could be avoided depending on the additional ingredients used to finish the product. Physicochemical characteristics should always be analyzed and considered for food product development, but sensory tests are also key to avoid investing time and resources when a new product is developed (e.g., imitating traditional methods). Considering the gastronomy culture for which the product was developed in the present study, the fermentation stage of the process could be avoided, reducing the number of unit operations and ingredients (starter), and optimizing the processing time (from 15 days to 1 day).

## Figures and Tables

**Table 1:** Sauce samples tested in the Napping^®^ test.

Code	Hot Sauce Type	Origin
200	Tabasco	USA
383	Tabasco chipotle	USA
274	Green tabasco	USA
958	Mexican green sauce	Mexico
394	Mexican red sauce	Mexico
664	Valentina	Mexico
060	Thai sriracha	Thailand
765	Spanish sriracha	Spain
491	Pasilla sauce	Denmark
587	Soya-based chilli sauce	Japan
332	Gochujang	South Korea
605	Kimchi	Japan
986	R3—sample	Spain

**Table 2:** Physicochemical and microbiological characteristics of the three green chili pepper mash samples.

Mash Samples (Time in Days)	pH	Sugars (mg g^−1^)			Log CFU/mL	
		Sucrose	Glucose	Fructose	TAC	TanC
R1-S (T15)	4.83 b	1.53 c	28.85 c	29.11 b	3.5 b	5.2 ab
R2-I (T15)	3.66 c	1.70 b	24.41 b	28.43 b	3.8 b	8.8 a
R3 (T0)	5.22 a	25.25 a	17.13 a	18.04 a	5.6 a	4.6 b
*p*-value	0.0001	<0.0001	0.001	<0.001	0.001	0.024

Note: Different letters within the column indicate different post hoc groupings by Tukey’s HSD (*p* ≤ 0.05).

**Table 3:** Instrumental color characteristics of the three different green chili pepper mash samples.

Mash Samples (Time in Days)	L*	a*	b*	Chroma	Hue (Rad.)	ΔE*
R1-S (T15)	43.45 b	2.45 a	35.67 b	35.75 b	1.50 b	11.92 a
R2-I (T15)	46.12 a	1.97 b	38.62 a	38.67 a	1.52 a	5.66 b
R3 (T0)	42.12 c	−7.87 c	33.02 c	33.95 c	1.34 c	-
*p*-value	<0.0001	<0.0001	<0.0001	<0.0001	<0.0001	<0.0001

Note: Different letters within the column indicate different post hoc groupings by Tukey’s HSD (*p* ≤ 0.05).

**Table 4:** Volatile composition of the mash samples expressed in relative abundance (% of total area).

Compound	RI (Exp)	RI (Lit)	R1-S (T15)	R2-1 (T15)	R3 (T0)	*p*-Value	Descriptor *
Hexanal	808	810	1.68	3.07	2.23	0.337	Fresh green, fatty, grass, leafy.
Disulfide, methyl 2-propenyl	922	920	4.59	5.02	5.03	0.862	Alliaceous, garlic, green, onion.
2-Heptenal	953	954	8.42	17.76	8.75	0.082	Pungent, green, vegetable.
**Dimethyl trisulfide**	963	976	**0.83 b**	**0.52 b**	**2.10 a**	**0.004**	Sulfureous, alliaceous, cooked.
1-Octen-3-ol	978	978	1.99	5.41	4.18	0.217	Sweet, mushroom, fungal, earthy.
**Limonene**	1034	1026	**19.65 a**	**7.21 b**	**1.03 b**	**0.007**	Citrus, range, fresh, sweet.
3-Octen-2-one	1044	1040	0.21	0.39	0.15	0.132	Earthy, spicy, herbal, sweet, mushroom.
Benzene acetaldehyde	1048	1045	0.55	0.27	0.31	0.516	Green, sweet, floral.
**2-Octenal**	1055	1056	**1.58 b**	**4.17 a**	**1.20 b**	**0.014**	Fatty, green, herbal.
2-Octen-1-ol	1065	n.d.	0.95	1.84	1.66	0.297	Green, vegetable.
1-Octanol	1069	n.d.	5.07	0.63	0.08	0.414	Waxy, green, orange.
Diallyl disulphide	1075	1087	12.70	16.45	14.25	0.714	Alliaceous, onion, garlic, metallic.
Allyl disulfide	1090	1087	3.94	1.97	1.81	0.248	Alliaceous, onion, garlic, metallic.
Allyl (*E*)-1-Propenyl disulfide	1096	n.d.	2.14	4.39	5.97	0.144	Sulfurous, alliaceous.
Linalool	1098	1095	3.07	1.37	n.d.	0.408	Citrus, floral, sweet.
Nonanal	1104	1102	4.47	0.31	0.62	0.442	Waxy, aldehydic, rose.
Allyl methyl trisulfide	1135	1135	8.42	9.51	16.04	0.171	Alliaceous, creamy, garlic, onion.
**2,6-Nonadienal**	1149	1154	**n.d. b**	**n.d. b**	**0.19 a**	**<0.0001**	Green, fatty, dry, cucumber.
**2-Nonenal**	1157	1162	**1.04 b**	**0.67 b**	**1.67 a**	**0.002**	Fatty, green, waxy, cucumber.
**2-Isobutyl-3-methoxypyrazine**	1173	1183	**n.d. b**	**n.d. b**	**0.28 a**	**<0.0001**	Green pea, green bell pepper.
**3-Vinyl-1,2-dithi-4-ene**	1181	1180	**2.82 b**	**1.99 b**	**10.96 a**	**<0.001**	n.d.
**Methyl salicylate**	1186	1190	**0.19 b**	**0.23 b**	**0.57 a**	**0.001**	Wintergreen mint.
**UNK.**	1188	n.d.	**2.11 ab**	**1.69 b**	**3.41 a**	**0.017**	n.d.
**UNK.**	1206	1214	**1.75 b**	**1.55 b**	**6.85 a**	**0.001**	n.d.
**2,4-Nonadienal**	1212	1215	**n.d. b**	**0.71 a**	**0.26 b**	**<0.001**	Fatty, melon, waxy.
**2-Decenal**	1261	1264	**1.74 ab**	**3.55 a**	**0.15 b**	**0.004**	Waxy, fatty, earthy, coriander.
Allyl trisulfide	1294	1300	7.97	7.28	7.55	0.869	Sulfurous, green, onion, garlic, metallic.
2,4-Decadienal	1312	1312	0.97	1.00	0.79	0.263	Fatty, oily, green.
Allyl trisulfide	1317	1300	0.40	0.19	0.54	0.383	Alliaceous, creamy, garlic, onion.
Eugenol	1340	1352	0.24	0.14	0.33	0.563	Sweet, spicy, clove, woody.
b-Phenylethyl butyrate	1398	1447	0.18	0.45	0.58	0.070	Musty, sweet, floral.
**Tetradecane**	1400	1400	**0.32 a**	**0.25 ab**	**0.15 b**	**0.029**	Mild, waxy.
**b-Chamigrene**	1473	1475	**n.d. b**	**n.d. b**	**0.30 a**	**<0.001**	n.d.

Legend: Retention indexes (RI), experimental (Exp) and found in literature (Lit). Bold letter to highlight the compounds significantly different among samples. Different letters within the row indicate different post hoc groupings by Tukey’s HSD (*p* ≤ 0.05). UNK: unknown compounds. * TGSC Information System [43].

**Table 5:** Main physicochemical characteristics of the sauce prototype.

	Mean	SD
pH	3.3	0.17
Sucrose (mg g^−1^)	0.85	0.18
Glucose (mg g^−1^)	16.96	3.51
Fructose (mg g^−1^)	17.50	3.51
L*	23.67	0.86
a*	19.37	1.63
b*	7.16	0.71
Chroma	20.65	1.76
Hue (rad.)	0.35	0.01

**Table 6:** Volatile composition of the final sauce sample (% of total area).

Compound	RI (Exp)	RI (Lit)	Mean (%)	SD	Descriptor *
Hexanal	802	810	0.21	0.01	Fresh green, fatty, grass, leafy.
3,4-Dimethylthiophene	903	888	0.23	0.13	Savory roasted onion.
Disulfide, methyl 2-propenyl	913	922	0.83	0.22	Alliaceous, garlic, green, onion.
a-Pinene	932	930	0.19	0.09	Dry, woody, resinous-piney.
2-Heptenal	952	954	2.69	0.51	Pungent, green, vegetable.
b-Myrcene	988	989	0.45	0.13	Woody, vegetative, citrus, fruity, tropical.
*p*-Cymene	1022	1031	0.49	0.16	Fresh, citrus, terpene, woody, spice.
Limonene	1027	1026	15.76	1.19	Citrus, range, fresh, sweet.
Benzene acetaldehyde	1038	1048	0.09	0.02	Green, sweet, floral.
1-Octanol	1069	1071	0.30	0.16	Waxy, green, orange.
Diallyl disulfide	1075	1087	6.69	1.72	Alliaceous, onion, garlic, metallic.
*o*-Guaiacol	1080	1088	0.38	0.11	Phenolic, smoky, spicy, medicinal.
Allyl (*Z*)-1-Propenyl disulfide	1089	1104	0.51	0.34	Sulfurous, alliaceous.
Allyl (*E*)-1-Propenyl disulfide	1096	n.d.	1.86	0.75	Sulfurous, alliaceous.
Linalool	1098	1098	2.41	1.53	Citrus, floral, sweet.
Nonanal	1103	1102	0.31	0.07	Waxy, aldehydic, rose.
Phenylethyl alcohol	1107	n.d.	0.14	0.01	Floral, rose, dried rose.
Fenchol	1114	1117	0.04	0.02	Camphor, pine, woody.
Allyl methyl trisulfide	1131	1135	2.76	1.51	Alliaceous, creamy, garlic, onion.
2-Nonenal	1157	1162	0.25	0.05	Fatty, green, waxy, cucumber.
Isoborneol	1165	1156	0.15	0.01	Balsam, camphor, herbal, woody.
Terpinen-4-ol	1176	1177	3.47	0.41	Musty, dusty.
3-Vinyl-1,2-dithiacyclohex-4-ene	1180	1180	1.26	1.16	n.d.
Methyl salicylate	1186	1190	0.31	0.15	Wintergreen mint.
a-Terpineol	1190	1197	1.04	0.25	Pine, terpene, lilac, citrus, woody.
Estragole	1193	1200	0.06	0.02	Sweet sassafras, anise, spice, green.
*p*-Cumic aldehyde	1238	1240	0.49	0.11	Spicy, cumin, green, herbal.
2-Decenal	1261	1264	0.64	0.25	Waxy, fatty, earthy, coriander.
*E*-Cinnamaldehyde	1267	1260	0.54	0.07	Sweet, spice, candy, cinnamon.
Ethyl guaiacol	1269	1280	0.43	0.12	Spicy, clove-like, medicinal.
Anethole	1281	1283	0.23	0.07	Sweet, anise, licorice, mimosa.
Isosafrole	1284	n.d.	0.78	0.09	Sweet, sassafras, spicy.
Allyl trisulfide	1293	1300	3.06	1.53	Sulfurous, green, onion, garlic, metallic.
a-Terpinyl acetate	1344	1347	0.48	0.09	Herbal, bergamot, lavender, lime, citrus.
Eugenol	1353	1353	35.33	3.26	Sweet, spicy, clove, woody.
Neryl acetate	1358	1366	0.62	0.11	Floral, rose, soapy, citrus, dewy pear.
Copaene	1372	1376	1.27	0.29	n.d.
Nerol acetate	1376	1365	0.14	0.08	Floral, rose, soapy, citrus, dewy pear.
Methyl eugenol	1396	1403	1.27	0.07	Sweet, spicy, clove, carnation, cinnamon.
Caryophyllene	1399	n.d.	0.59	0.19	Sweet, woody, spice, clove.
*E*-b-Caryophyllene	1415	1416	7.54	0.63	Spicy, clove, woody, nut skin.
Humulene	1450	1452	0.85	0.24	Woody, oceanic-watery, spicy-clove.
a-Muurolene	1493	1500	0.14	0.01	Herbal, woody, spicy.
b-Bisabolene	1504	1509	0.42	0.11	Balsamic, citrus, myrrh, spicy.
Myristicine	1513	1516	1.85	0.13	Spicy, warm, balsamic, woody.
Elemicin	1542	1547	0.45	0.03	Spice, flower.

Legend: Retention indexes (RI), experimental (Exp) and found in literature (Lit). * TGSC Information System [43].

## Data Availability

The data used to support the findings of this study can be made available by the corresponding author upon request.
